# Global Human Consumption Threatens Key Biodiversity Areas

**DOI:** 10.1021/acs.est.2c00506

**Published:** 2022-05-05

**Authors:** Zhongxiao Sun, Paul Behrens, Arnold Tukker, Martin Bruckner, Laura Scherer

**Affiliations:** † Institute of Environmental Sciences (CML), Leiden University, 2333 CC Leiden, the Netherlands; ‡ College of Land Science and Technology, China Agricultural University, 100193 Beijing, China; § Leiden University College The Hague, 2595 DG The Hague, the Netherlands; ∥ The Netherlands Organisation for Applied Scientific Research TNO, 2595 DA The Hague, the Netherlands; ⊥ Institute for Ecological Economics, Vienna University of Economics and Business, 1020 Vienna, Austria

**Keywords:** biodiversity loss, countryside species−area relationship, multiregional input−output analysis, international trade, land-use intensity

## Abstract

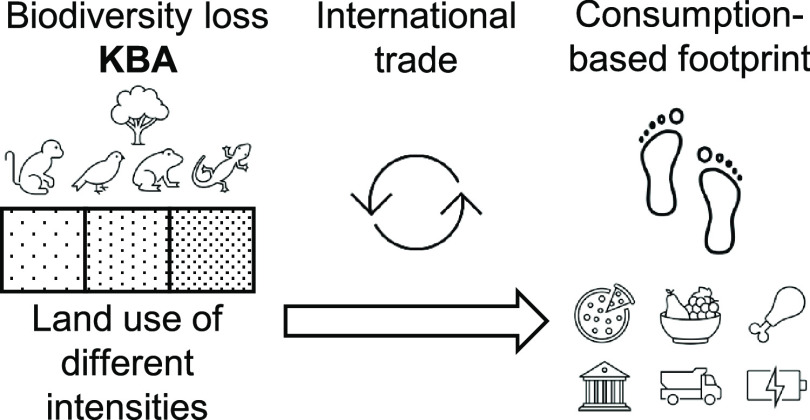

Key biodiversity areas (KBAs) are critical regions for preserving global biodiversity. KBAs are identified by their importance to biodiversity rather than their legal status. As such, KBAs are often under pressure from human activities. KBAs can encompass many different land-use types (e.g., cropland, pastures) and land-use intensities. Here, we combine a global economic model with spatial mapping to estimate the biodiversity impacts of human land use in KBAs. We find that global human land use within KBAs causes disproportionate biodiversity losses. While land use within KBAs accounts for only 7% of total land use, it causes 16% of the potential global plant loss and 12% of the potential global vertebrate loss. The consumption of animal products accounts for more than half of biodiversity loss within KBAs, with housing the second largest at around 10%. Bovine meat is the largest single contributor to this loss, at around 31% of total biodiversity loss. In terms of land use, lightly grazed pasture contributes the most, accounting for around half of all potential species loss. This loss is concentrated mainly in middle- and low-income regions with rich biodiversity. International trade is an important driver of loss, accounting for 22–29% of total potential plant and vertebrate loss. Our comprehensive global, trade-linked analysis provides insights into maintaining the integrity of KBAs and global biodiversity.

## Abstract 2

Global land use threatens biodiversity within key biodiversity areas (KBAs). We find a much higher proportional level of biodiversity loss within KBAs than in other areas.

## Introduction

Biodiversity loss severely alters and threatens ecosystem functioning, and human-driven land use is the largest threat to terrestrial biodiversity.^1,2^ This land use has led to a rapid acceleration in the rate of species extinction, far exceeding the estimated planetary boundaries.^3−5^ The urgency for biodiversity protection is reflected in international agreements, for instance, in Sustainable Development Goals (SDGs) 14 and 15^6^ and the previous 2020 Aichi Biodiversity Targets.^7^ Recent developments in biodiversity protection include the identification of key biodiversity areas (KBAs), sites that significantly contribute to the global persistence of biodiversity.^8^ KBAs reflect an increasing appreciation of the complexities required to maintain biodiversity and are identified based on 11 globally standardized threshold-based criteria within five categories: threatened biodiversity, geographically restricted biodiversity, ecological integrity, biological processes, and irreplaceability. Around 16,000 KBAs have been identified as of 2020 (Figure S5),^9^ and they are likely to play a more central role in the main framework for identifying future conservation priorities.^10−12^ This approach contrasts with other methods that generally address one biome or a group of species, thereby omitting important biodiversity integrity.^13^ Even though KBAs play an important role in biodiversity protection, little is known about the biodiversity loss driven by land use within KBAs.

KBAs encompass regions of human activities and land use. However, it is not only the amount of land use that drives biodiversity loss but also the intensity of that land use.^14,15^ To investigate the impacts of land use on biodiversity, researchers have used characterization factors (CFs) derived from the countryside species–area relationship (SAR) (see the Materials and Methods section).^14,15^ These CFs estimate the potential species extinctions driven by a unit of land use if it remains in its current state over the long term.^14,15^ Land use causing habitat loss can lead to species extinctions both immediately and over the long term (also known as extinction debt).^16^ The SAR is insensitive to such timing differences between the short and long term. As such, the SAR is widely used to estimate species extinctions due to habitat loss over the long term.^14−16^ Although land use is a local phenomenon, the CFs also evaluate if a species faces the potential for loss globally and will therefore go extinct.^15^ Here, we refer to global species-equivalents potentially lost over the long term as *species lost* and use this approach in our analysis.^15^

Due to increasing levels of globalization, local human land use is often driven by global demand, which enhances the geographic disconnection between producers and consumers as supply chains grow in complexity. For example, biofuels consumed in the EU can drive loss in Indonesia when these fuels are derived from palm oil.^17^ Previous estimates have concluded that 25% of global species lost^14^ and 30% of global species threats^18^ are driven by international trade, a larger proportion than for estimates of several other trade-based displacements such as carbon emissions.^19^ The displacement of biodiversity loss is generally from high-income to middle- and low-income nations.^20^ As such, assessments of the responsibility for land use in KBAs benefit from taking both production-based (responsibility is shouldered by the producing nation) and consumption-based (responsibility is shouldered by consumers of products all along the value chain) perspectives.

A previous analysis found that global cropland, even inside protected areas, has large impacts on vertebrate species, but did not include the role of other land uses, impacts on other species, or the responsibility of international trade.^21^ There have been efforts to map biodiversity loss in trade. For instance, Moran et al. (2017) mapped consumption-based global biodiversity loss hotspots but did not identify biodiversity loss due to a specific driver (e.g., land use) and used highly aggregated sectors for the economic activities driving this loss.^20^ Other studies have traced biodiversity loss along the global supply chain for some products back to specific production locations (e.g., the Brazilian Cerrado) but have not examined the global picture.^22^

KBAs are critical regions in efforts to preserve global biodiversity. Understanding the issues faced within KBAs is crucial to developing appropriate policies for regions that have been identified to be of key importance. Despite this importance, the biodiversity within KBAs is still threatened by both the amount and intensity of land use. It is unclear how many species are expected to go extinct over the long term if the current land use continues. In addition, human consumption is the underlying driver of global land use. Therefore, here, we provide a global, trade-linked assessment of biodiversity loss within KBAs. We examine the potential global loss of terrestrial species driven by domestic and teleconnected land use (i.e., land use driven by the consumption of imported goods and services) both within and outside KBAs (to provide a comparison of activities within and outside KBAs). We do this by building a hybrid model using physical and monetary input–output databases, spatially explicit land use maps, and CFs of biodiversity loss (see the Materials and Methods section for further details). Spatially explicit information on biodiversity loss within KBAs linked to trade can help all agents along global value chains to cooperate on solutions for targeted biodiversity conservation.

## Materials and Methods

We assess global biodiversity loss in 2005 driven by anthropogenic land use within KBAs by combining multiregional input–output (MRIO) analysis with spatial analysis (Figure S1). Using MRIO analysis, we link production and associated environmental pressures to consumption anywhere in the world at the national scale (see section MRIO Analysis below). Then, we allocate the consumption-based land use of a specific country into grid cells with the help of global land-use maps and assign land-use intensities. Different land-use types and intensities determine the potential biodiversity loss at a location per area of land use, reflected by characterization factors (CFs). The biodiversity loss within the boundaries of KBAs can be delineated via this spatially explicit information. In short, we calculate biodiversity loss driven by land use both within KBAs and outside KBAs to provide a comparison. We focus on biodiversity loss within KBAs in the Results section (see section Deriving Spatially Explicit Biodiversity Loss Related to Land Use below).

### MRIO Analysis

The starting point for quantifying biodiversity loss within KBAs is gridded land-use data (see the next section). This enables a link to CFs on biodiversity loss per m^2^ of land use (Figure S1). While agriculture sectors dominate human land use, traditional global MRIO databases have highly aggregated agricultural sectors or regions. This is addressed using the recently developed food and agriculture biomass input–output (FABIO) table, a consistent, balanced, physical input–output database based on FAOSTAT data, covering 192 countries/regions and 128 agriculture, food, and forestry products^23^ (excluding nonagricultural sectors). To cover nonagricultural sectors, we build an integrated model framework linking FABIO and EXIOBASE for the year 2005 (Figure S1). EXIOBASE v3.6 is a highly detailed, monetary global multiregional input–output database, including 200 products and 49 countries or regions.^24^ EXIOBASE covers nonagricultural sectors in detail, and by combining the two MRIO databases, we can harness the advantages of both. An *other uses* matrix (*Z*_other_ in Figure S1) links FABIO with EXIOBASE by providing agriculture and forestry biomass inputs in physical units for manufactured products in monetary units. We consider land use for food consumption (***y***_**FABIO**_) and nonfood consumption (***y***_**EXIO**_) separately. To attribute land use to consumers across countries, we use a spatially explicit multiregional input–output (SMRIO) model^17,25^ (eqs 1 and 2). You can see details of constructing the integrated FABIO and EXIOBASE framework at https://github.com/fineprint-global/fabio-hybrid.

SMRIO connects the economic sectors in a standard MRIO database with spatially explicit estimates of environmental pressures (e.g., land use) to track a country’s final consumption to the location of the embodied environmental pressures.^25^ The SMRIO in the study is used to estimate the impact of the demand for a given commodity (e.g., palm oil) in a specific region or country (e.g., the US) through land use in a region or country (e.g., Indonesia) on a species group (e.g., plants). We assume a proportional approach in the SMRIO. That is, we assume an equal spatial distribution of land use driven by the consumption of country *s* across all grid cells within producing country *r* of the corresponding land-use type and intensity. The full model is expressed mathematically as
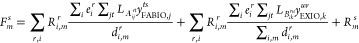
1
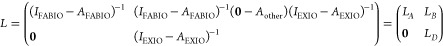
2where *F*_*m*_^*s*^ is the global spatial distribution of land use for land-use type and intensity *m* driven by the final consumption of country *s* for both FABIO and EXIOBASE. *R*_*i,m*_^*r*^ defines the spatial distribution of land use, represented in absolute values, for product *i* (e.g., cropland for different crops) produced in country *r* under land-use type and intensity *m*. *R*_*m*_^*s*^ defines the spatial distribution of land-use type and intensity *m* due to final consumption of product *i* in country *s*, represented in absolute values. The spatial distribution of land use for each product *i* under land-use type and intensity *m* is described in the Supporting Methods and Tables S1–S4. *e*_*i*_^*r*^ is the environmental intensity (land-use area per unit of output) of product *i* in the producing country *r*. ***y***_FABIO*,j*_^*ts*^ indicates the final consumption of FABIO product *j* in country *s* that originates from country *t*, which is the last country exporting to country *s* in FABIO (that is, in a supply chain of four countries producer A, intermediate B, intermediate C, and consumer D, this refers to country C). ***y***_EXIO*,k*_^*uv*^ indicates the final consumption of EXIOBASE product *k* in country *v* that originates from country *u*, which is the last country exporting to country *v* in the other uses matrix (i.e., the required amount of biomass inputs per euro of manufactured product) in Figure S1. Since EXIOBASE has a higher spatial aggregation (with five “rest of world” regions), we assume the same per-capita consumption for FABIO countries, which fall under the five “rest of world” regions in EXIOBASE (see the mapping relationship in Table S5). *d*_*i,m*_^*r*^ expresses the total land use of product *i* in country *r* under land-use type and intensity *m.* Since the matrix of technical coefficients (i.e., input requirements per unit of output) is a block matrix integrating FABIO and EXIOBASE, we can derive the Leontief inverse ***L*** via a simplified eq (2) using ***L***_***A***_ and ***L***_***B***_ as the subcomponents of the inverse in eq (1). ***I***_FABIO_ is the identity matrix with the same dimension of FABIO, and ***I***_EXIO_ is the identity matrix with the same dimension as EXIOBASE.

*Z*_FABIO_ is a 24,576 (192 countries or regions × 128 products) rows × 24,576 columns matrix that describes the input–output relationship between agriculture, food, and forestry products within and among nations. The physical units of *Z*_FABIO_ are derived from FAOSTAT and depend on the products. The physical units of live animals and forestry products are *heads* and *m^3^*, respectively. The remaining agriculture and food products are measured in *tonnes*. The total output vector ***x***_**FABIO**_ has 24,576 elements and uses the same units as the abovementioned categories. The technical matrix of FABIO (*A*_FABIO_) is calculated by the equation *A*_FABIO_ = *Z*_FABIO_*x̂*_FABIO_^–1^. *Z*_EXIO_ is a 9800 (49 countries or regions × 200 products) rows × 9800 columns matrix that describes the input–output relationship between products within and among countries or regions in EXIOBASE. The monetary unit of *Z*_EXIO_ is euros. The total output vector ***x***_**EXIO**_ has 9800 elements and is also measured in euros. The technical matrix of EXIOBASE (*A*_EXIO_) is calculated by the equation *A*_EXIO_ = *Z*_EXIO_*x̂*_EXIO_^–1^. *Z*_other_ is a 24,576 rows × 9800 columns matrix that describes the volumes of agricultural sectors in FABIO as input to economic sectors in EXIOBASE. The physical units of *Z*_other_ are the same as those of the sectors in FABIO. *A*_other_ is calculated by the equation *A*_other_ = *Z*_other_*x̂*_EXIO_^–1^. It has the same dimensions as FABIO in rows (24,576) and EXIOBASE in columns (9800).

### Deriving Spatially Explicit Biodiversity Loss Related to Land Use

To quantify global species loss driven by human land use at different land-use intensities, we use the latest characterization factors (CFs) developed by Chaudhary & Brooks, which represent the year 2005.^15^ The CFs allow for an estimation of potential global extinctions driven per unit of land use.^15^ The CFs were derived from the countryside species–area relationship (SAR) for regional species loss of 804 terrestrial ecoregions.^15^ Ecoregions are defined based on their biodiversity, habitat diversity, and environmental properties, and thus delineate biologically similar areas.^26^ Based on this, in estimating the spatially explicit biodiversity loss driven by global land use, we assume that the value of CFs in each pixel is the same for all pixels situated within the ecoregion (as also assumed by others, including in ref (27)). Regional species loss was subsequently multiplied with a vulnerability score of taxa based on species’ geographic ranges and threat levels from the International Union for Conservation of Nature (IUCN) Red List to estimate global species loss.^15^ The vulnerability score is 1 if all species within a region are “critically endangered”, as assessed by the IUCN Red List, and have their entire range inside that region (i.e., they are strictly endemic to that region). Thus, local land use within KBAs can potentially lead to global species extinctions, especially if the species is endemic and critically endangered. The unit is the *potential global species loss* (referred to as species lost) per *m^2^*.

The CFs consider five taxa (mammals, amphibians, reptiles, birds, and plants) and five land-use types (managed forest, plantation, pasture, cropland, and urban) under three intensity levels (minimal, light, and intense) for terrestrial ecoregions.^15^ Specifically, each taxon consists of numerous species, including about 5490 mammals, 6433 amphibians, 9084 reptiles, 10,104 birds, and 321,212 plants, as indicated for its predecessor method.^28^ We use average instead of marginal CFs. Marginal CFs apply to only small changes from the current situation (e.g., one additional m^2^ of land use), whereas average CFs apply to the average of larger changes from the current situation.^28^ In this study, we are investigating large changes from natural habitat to the current land use pattern in KBAs or even globally. After computing the spatial distribution per unit area of each land-use type at different land-use intensities driven by final consumption in a given region, we multiply the corresponding CFs with consumption-based land-use data to obtain consumption-based global species loss for each taxon (eq 3).
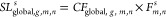
3

*SL*_global,*g,m,n*_^*s*^ is the potential global species loss for taxon *g* for land-use type and intensity *m* in grid cell *n* driven by final consumption in country *s*. *CF*_global*,g,m,n*_ is the land occupation *CF* (species lost per unit land use) for taxon *g* for land-use type and intensity *m* in grid cell *n*. *F*_*m,n*_^*s*^ is the land use for land-use type and intensity *m* in grid cell *n* driven by final consumption in country *s*. *F* is derived from eq 1.

After finding the global distribution of biodiversity loss driven by human consumption, we use KBA boundaries^9^ to get the subset of biodiversity loss from land use within KBAs. The consumption-based biodiversity loss is the sum of agriculture- (and forestry-) related biodiversity loss (from FABIO) and non-agriculture-related biodiversity loss (from EXIOBASE) (Figure S1).

We distinguished four variables determining the magnitude of biodiversity loss due to land use (i.e., area of KBAs, share of KBAs with anthropogenic land use, species richness per area of land use within KBAs, potential relative global species loss). To keep the spatial scale uniform in our analysis, we aggregate the values of these variables within KBAs to the country level.
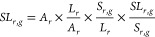
4where *SL*_*r,g*_ is the potential global species loss within KBAs due to land use for taxon *g* in country *r*; *A*_*r*_ is the area of KBAs in country *r*; *L*_*r*_ is the land-use area in country *r*; and *S*_*r,g*_ is the species richness for occupied land use within KBAs for taxon *g* in country *r* (assuming that the number of species per area unit is the same within an ecoregion). To estimate the contribution of each variable, we used contribution to variance (CTV) analysis as in previous studies.^29,30^ We calculated CTV based on Spearman’s rank correlation coefficients (*R*) between species loss and its variables (*d*) due to the non-normality of species loss and its variables.
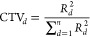
5

## Results

### Global Picture of Biodiversity Loss from Land Use within KBAs

**Figure 1:**
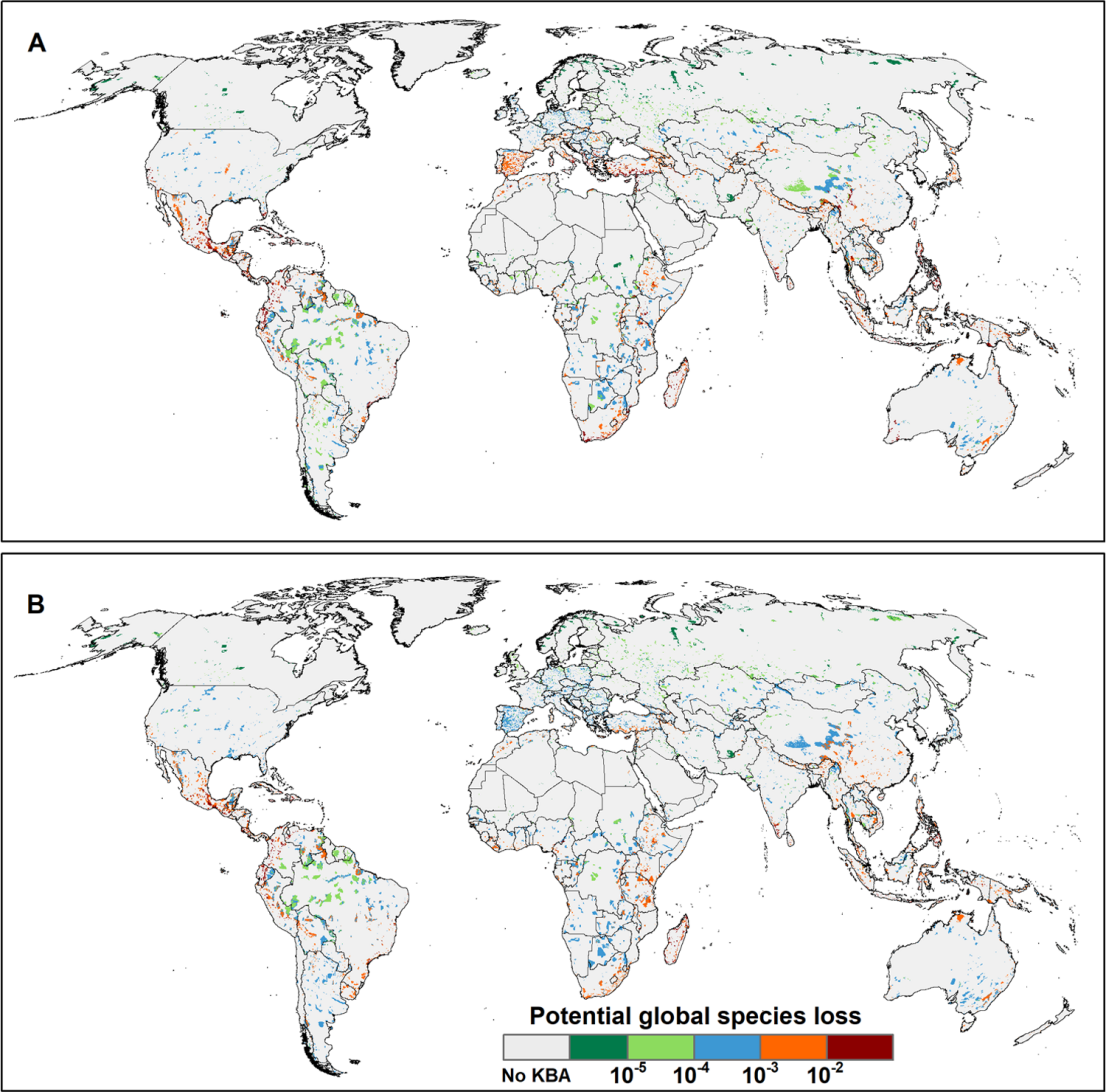
Potential global species loss driven by land use within KBAs for (A) plants and (B) vertebrates (mammals, birds, amphibians, and reptiles). The results are the sum of potential global species loss across all land-use types and intensities driven by consumption of countries.

Overall, we find that human land use within KBAs leads to a total potential loss of 781 terrestrial plant species (hereafter referred to as plants) and 208 terrestrial vertebrate species, including mammals, birds, amphibians, and reptiles (hereafter referred to as vertebrates) (Figure 1). Here, we report the aggregated category of vertebrates and plants for ease of communication. Interested readers that would like further information on vertebrates (mammals, birds, reptiles, and amphibians) may like to consult the SI, where we provide results per vertebrate class. The loss accounts for 0.2% of global plant species and 0.7% of global vertebrate species. To put these results on land use within KBAs in perspective compared to total land use, our results suggest that total land use (inside and outside KBAs) causes a potential loss of 5038 plant species and 1765 vertebrate species (Figure S2). The loss contributes to 1.6% of global plant species and 5.9% of global vertebrate species. The proportion is similar to multiple previous global studies that focus on species threatened by land use.^14,31,32^ While land use within KBAs only accounts for 7% of total land use, it drives 16% of the potential global plant loss and 12% of the potential global vertebrate loss compared to total land use. The biodiversity loss due to land use differs among regions (Figure S4), since different regions have different mixes of land-use types, varying land-use intensities (we cover minimal, light, and intensive land-use patterns here), consume different goods, and have different levels of biodiversity. Light use of pasture within KBAs is the primary driver of biodiversity loss, accounting for a potential loss of 382 plant species (49% of losses) and 91 vertebrate species (44% of losses). This is because pasture with light use accounts for the largest proportion (50%) of land use within KBAs (Figure S4). Pasture also sometimes displaces species-rich natural ecosystems, such as tropical forests in Latin America,^33^ thereby causing severe biodiversity loss. The exact mechanism by which cattle grazing influences biodiversity varies depending on location and management practices, but in general, biomass removal, trampling and destruction of root systems, and competition between livestock and wildlife have the largest impacts on reducing biodiversity.^33,34^

At a regional level, there are several distinct biodiversity loss hotspots. Plant loss is highly concentrated across Mexico, the nations of Central America, the Caribbean, Colombia, Venezuela, Madagascar, Southern Europe, South Africa, the southern part of India, the southwestern part of China, Southeast Asia, and the southwestern and southeastern parts of Australia (Figure 1). Vertebrate loss from land use within KBAs is also mainly located in Mexico, the nations of Central America, the Caribbean, Colombia, Venezuela, Madagascar, southern India, and Southeast Asia (Figure 1).

We decompose the biodiversity loss within KBAs for each country into four variables determining its magnitude, namely: (1) the area of KBAs, (2) the share of KBAs with anthropogenic land use, (3) the species richness per unit of land use within KBAs, and (4) the potential relative global species loss (Figures S6–S9). We find that these four variables all significantly contribute to biodiversity loss (Table S13). For both plant loss and vertebrate loss, relative species loss contributes the most with 54 and 44%, respectively (Table 1). This means that the fraction of species lost strongly influences the absolute species loss. The area of KBAs is the second-largest driver of species loss, contributing to 21 and 40% of plant and vertebrate loss within KBAs, respectively. Naturally, the larger the KBA area in a country, the more likely land use in that country has an impact on biodiversity in KBAs. For plant loss, the species richness per unit of land use has almost the same contribution as the area of KBAs. The more plant species there are, the more species can get lost by occupying the land. This variable has a milder impact on vertebrates. The share of KBAs with anthropogenic land use has the least impact on species loss, accounting for 6 and 7% of plant loss and vertebrate loss, respectively.

**Table 1:** Contributions to Variance of Potential Species Loss (%) per Variable at the Country Level

variable	plant species loss	vertebrate species loss
area of KBAs	21	40
share of KBAs with anthropogenic land use	6	7
species richness per area of land use within KBAs	19	9
potential relative global species loss	54	44

### Biodiversity Loss from Different Land-Use Types with Three Intensities

**Figure 2:**
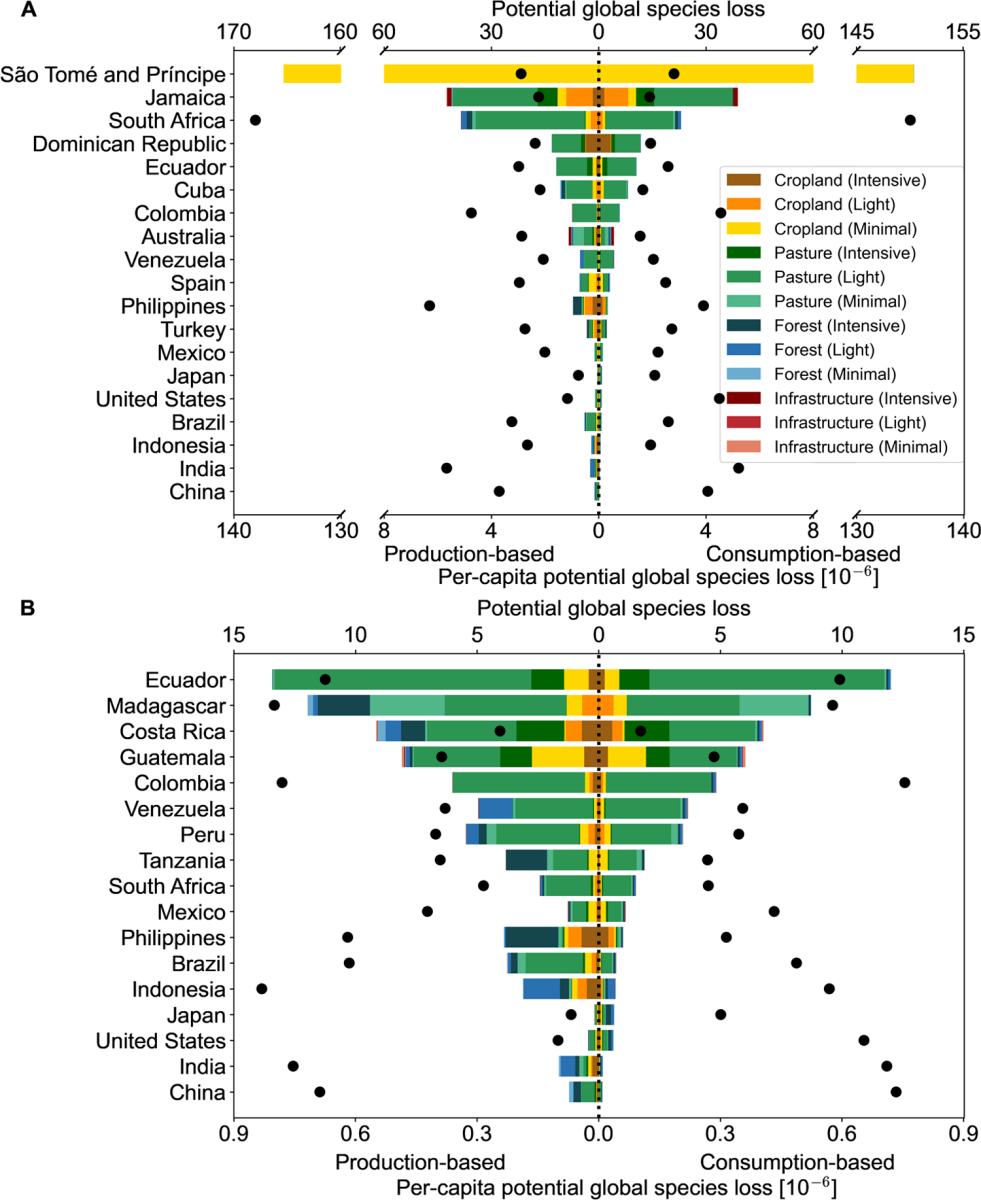
Potential global species loss from land use within KBAs for (A) plants and (B) vertebrates (mammals, birds, amphibians, and reptiles). On the *x*-axis, the production-based perspective is shown to the left of zero and the consumption-based perspective to the right. The *y-*axis lists the top 15 countries/regions with the largest consumption-based or production-based biodiversity loss from land use within KBAs at the national level. Since there are some differences in the top 15 from the two perspectives, the overall number is larger than 15. The bars show the per-capita values of biodiversity loss within KBAs per land-use type and intensity. The points show the total national biodiversity loss with a value shown by the upper *x*-axes on top of each plot. Forest includes managed and planted forests.

Top countries with the largest consumption-based or production-based biodiversity loss from KBAs are the major contributors to global biodiversity loss within KBAs (Figure 2). For example, the top 15 countries with the largest consumption-based or production-based biodiversity loss from KBAs account for 62–73% of total plant or vertebrate loss from either a production or consumption perspective. Consumption-based biodiversity loss from land use within KBAs ranks the highest in biodiverse regions, such as South Africa and Madagascar (i.e., mainly as a result of domestic consumption), as well as in areas that import large amounts of loss via trade (e.g., the US). For plant species, South Africa sees the largest loss from a consumption- and production-based perspective (149 and 168 species lost from land use within KBAs, respectively). Pasture with light use is the primary land-use driver in South Africa, contributing to 82 and 80% of consumption- and production-based plant loss, respectively.

São Tomé and Príncipe sees the largest potential per-capita plant loss (i.e., national plant loss divided by the country’s population) from a consumption- and production-based perspective (both 135 × 10^–6^ species lost per capita from land use within KBAs). This is almost entirely due to land used for crops at a minimal use intensity. Such a large result is driven by São Tomé and Príncipe’s position as an important region for endemic species and more than half of its land area being covered by KBAs, a higher share than any other country.^35^ There is a large drop in potential per-capita plant loss in the next most prominent country, South Africa, at 3 × 10^–6^ and 5 × 10^–6^ consumption- and production-based species loss per capita, respectively.

Focusing on potential vertebrate loss, Colombia’s teleconnected land use within KBAs drives the largest consumption-based loss (13 species lost), where pasture contributes to 89% of the potential loss. In contrast, Indonesia sees the largest production-based impacts, with 14 species lost from land use within KBAs. Here, managed and planted forests are the main drivers, contributing 61% of the potential loss. When looking at land use also outside KBAs, Brazil and the US surpass Indonesia and Colombia, causing the largest production- and consumption-based total potential vertebrate species loss, respectively (Figure S3). Among the top countries (Figure 2), Ecuador sees the largest per-capita consumption-based and production-based potential vertebrate loss (0.7 × 10^–6^ and 0.8 × 10^–6^ species lost from land use within KBAs), where pasture with light use accounts for 80 and 79%, respectively.

### Biodiversity Loss Embodied in International Trade

**Figure 3:**
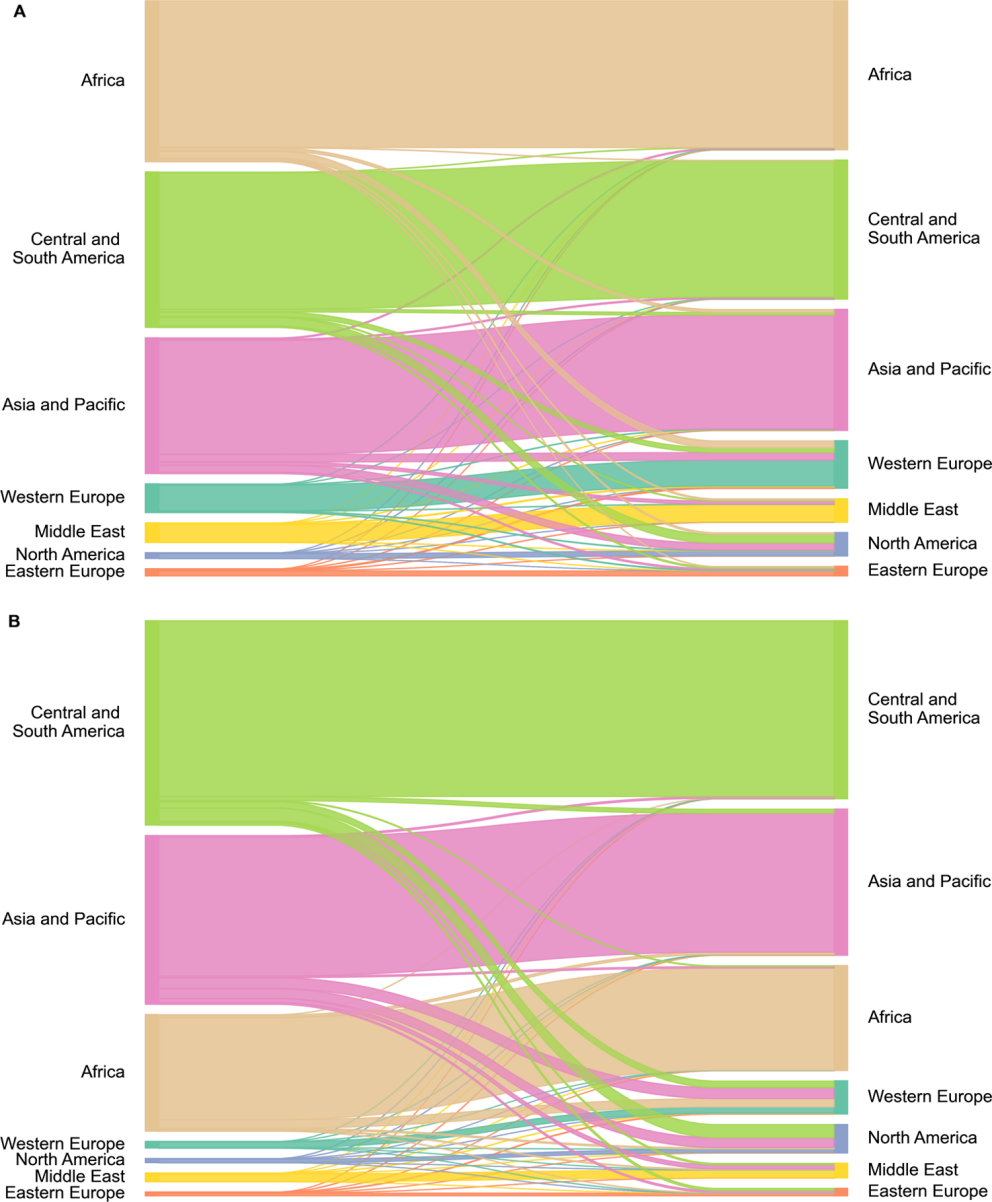
Embodied biodiversity loss flows for (A) plants and (B) vertebrates (mammals, birds, amphibians, and reptiles) from land use within KBAs. Producing regions are on the left of the figure, consuming regions on the right. Regions are ordered by the magnitude of loss in the consuming region. The width of the flows is proportional to the magnitude of the potential global species loss.

International trade is a major driver of biodiversity loss, contributing around a third of potential global vertebrate loss and a quarter of plant loss within KBAs (Figure 3). To illustrate flows from regions where biodiversity loss occurs to regions that consume the goods produced, we aggregate countries/regions into seven world regions. Western Europe and North America drive the largest biodiversity loss embodied in international trade (Figure 3). For instance, 79% of consumption-based potential plant loss in North America is driven by international markets, mainly from Central and South America (37%) and Asia and Pacific (30%) (Figure 3). Similarly, 82% of consumption-based potential vertebrate loss in Western Europe is embodied in international trade, mainly from Asia and Pacific (33%), Africa (26%), and Central and South America (20%) (Figure 3). This is similar to other studies finding that Western Europe and North America were responsible for 69% of biodiversity impacts transferred through international trade.^14^ Specifically, the largest flow of potential plant loss via trade (excluding domestic production and consumption) is from the Philippines to the US, with 2.4 species lost from land use within KBAs. In contrast, the largest flow of potential vertebrate loss through trade is from Indonesia to the US, with 1 species lost. The US is involved in 7 and 6 of the top 10 trade flows for vertebrates and plants, respectively.

### Biodiversity Loss Driven by the Consumption of Products

**Figure 4:**
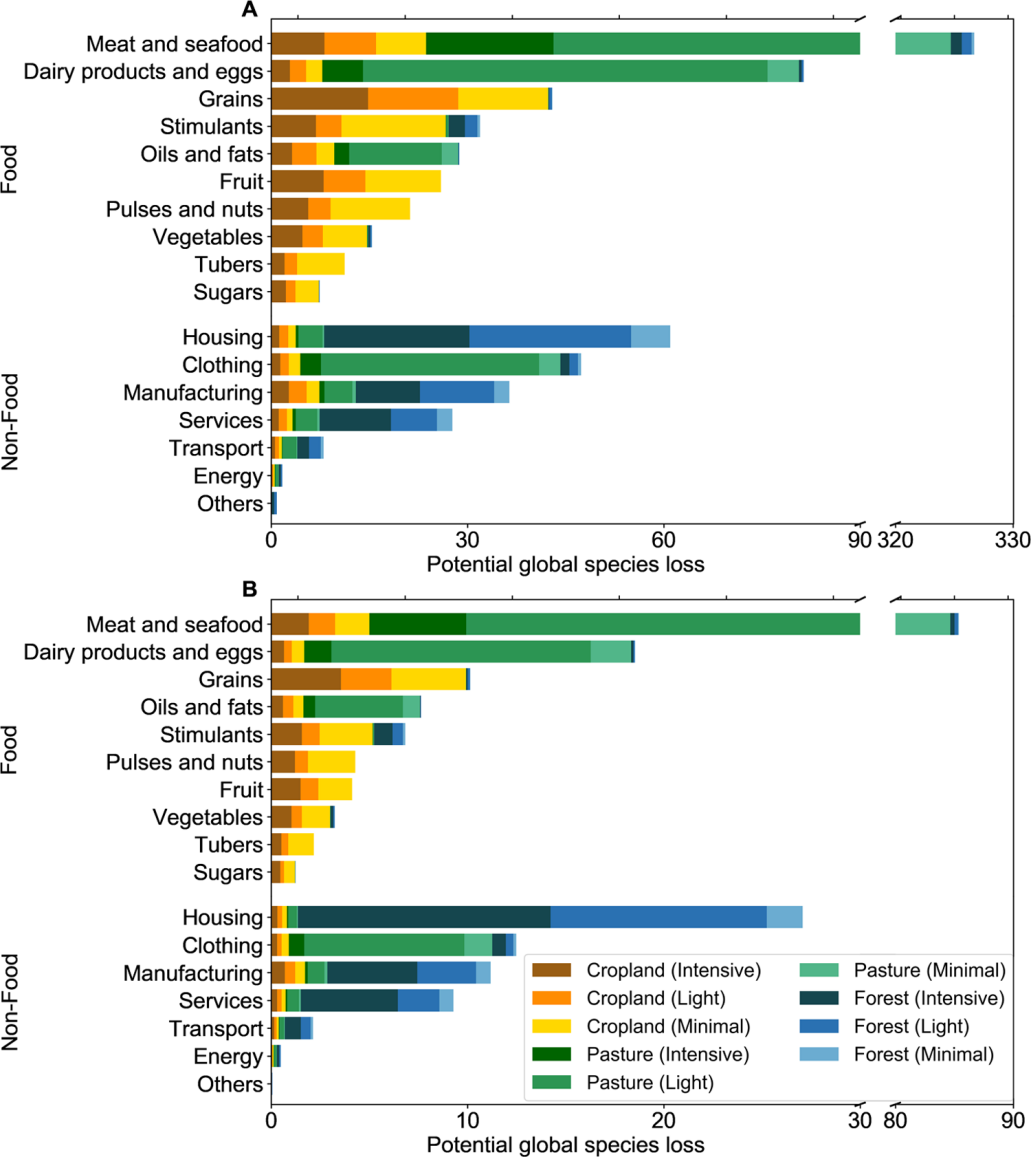
Potential global species loss due to specific product consumption from land use within KBAs for (A) plants and (B) vertebrates (mammals, birds, amphibians, and reptiles). Forest includes managed and planted forests.

Overall, food products contribute 74% of biodiversity loss within KBAs, with the remaining 26% driven by non-food products (Figure 4). Food-driven biodiversity loss is dominated by the consumption of animal products, which account for more than half of the total biodiversity loss within KBAs, with 408 plants (52%) and 104 vertebrates lost (50%). Within this, the consumption of bovine meat is the largest single contributor to biodiversity loss, with 241 plants lost (31%) and 63 vertebrates lost (30%). The result is consistent with Marques et al., who found that cattle farming was the largest driver of bird species loss from 2000 to 2011.^14^ Since they did not consider land-use intensity, we can further clarify that this is more due to the extent of cattle farming than its intensity compared to other land uses. In addition, feeding livestock uses large areas of land. For example, 60% of land use within KBAs is pasture which is used for livestock ranching. Further, around 30% of cropland within KBAs is used to feed livestock.

The next largest sector is housing (Figure 4), which includes all built infrastructure (e.g., roads), with 61 plants lost (8%) and 27 vertebrates lost (13%), driven mainly by “Construction work” and “Furniture” sub-categories, both of which heavily rely on forest products. Clothing contributes a further 6%, mainly driven again by pasture for animal products such as leather.

## Discussion

We provide a comprehensive overview of global, land-use driven biodiversity loss within and outside KBAs by (1) using potential global species loss for multiple taxa rather than a single aggregated index,^14,36^ (2) considering different land-use intensities rather than just one,^27^ and (3) analyzing the effect of international trade on biodiversity loss rather than production-based biodiversity loss.^15^ We find that pasture is the largest contributor to biodiversity loss from land use within KBAs, with 58% of total potential plant species loss and 56% of vertebrate species loss (Table S9). Consequently, animal products are the primary drivers of biodiversity loss, in particular bovine meat. Lowering the consumption of animal products could reduce agricultural expansion and thus lead to land sparing, whereas a reduction in land-use intensity could lead to land sharing, which could potentially reverse biodiversity declines.^37,38^

We estimate that a quarter of global plant losses and a third of global vertebrate losses are embodied in international trade. This is slightly higher than a previous estimate of 20% based on net primary productivity in biodiversity hotspots^39^ and similar to previous estimates of 25% for global endemic vertebrate loss^40^ or 30% for threats to vertebrates.^18^ In the international market, high-income nations can outsource land use and the associated biodiversity loss to other middle- and low-income nations that may have lower regulatory standards and higher biodiversity.^14,21^ These differences partly drive leakage in biodiversity loss through international trade (analogous to carbon leakage). For example, Europe restored territorial forests by 9% (∼13 Mha) while outsourcing 11 Mha deforestation due to crop displacement from 1990 to 2014.^41^ This deforestation occurs in many biodiversity-rich regions.^41^ These dynamics may change in the future, as agricultural development is projected to grow due to rapidly increasing population and per-capita income in tropical and subtropical regions, which may result in higher local consumption and lower exports.^37^

The estimation of biodiversity loss would vary with land-use change. For example, the increasing population and economic growth, by changing consumption patterns (e.g., increasing animal product consumption), especially in rapidly growing regions, caused land-use expansion and, therefore, more biodiversity loss.^14^ Our estimation is based on land use in the year 2005. As such, land-use change since 2005 may have caused further biodiversity loss within KBAs, leading to a possible underestimate in our results. For example, pasture decreased by 0.2%, while cropland and urban area increased by 5.8 and 13.2% within KBAs from 2005 to 2019.^42^ These proportions are higher than the global average change,^42^ and this means that human activities are increasingly threatening global biodiversity, given the importance of KBAs for the global persistence of biodiversity.

The formal status of KBAs is uncertain and appropriate metrics to assess progress toward reaching biodiversity protection goals within them are needed. It is possible to argue that KBAs are both either more or less exploited than neighboring regions. They might be more exploited because they provide more resources, such as food, timber, and fiber,^43,44^ but also more protected because 56% of global terrestrial KBAs are in protected areas, much higher than the global average level of protected areas (14%).^45^ Protected areas are established to prevent habitat loss and slow biodiversity decline. Coverage of KBAs by protected areas can be used to measure the progress toward their protection.^46^ However, the status of a protected area does not guarantee adequate management.^12^ For example, cropland within protected areas causes 18% of total species threats of global cropland.^21^ In addition, protected areas can also have little biodiversity conservation value, while KBAs are important for the persistence of biodiversity.^12^ Alternative metrics may include the relative change of the current value compared to a reference value for different biodiversity and habitat indicators within KBAs.^12^ This reference value might be the expected biodiversity in a region if there were little or no human disturbance. These metrics need extensive data from monitoring systems (e.g., remote sensing, in situ monitoring, and others).^12^

There are several opportunities to reduce uncertainties in future research. Given the dominance of land use for food systems, the first set of opportunities arises from improved agricultural mapping. Advances in remote sensing^47,48^ and the use of crowdsourced data^49^ may improve the accuracy of crop- and animal-specific maps, thereby enabling a more accurate link between land-use pressures and biodiversity loss. In terms of assessing biodiversity loss, improving the resolution of CFs can reduce uncertainties. Although other studies employ this same assumption to study biodiversity loss at a grid cell level,^27^ it would be an improvement to develop biodiversity CFs in line with the resolution of land use (i.e., 5 arcmin in this paper). A SAR approach could lead to both over-^50^ or underestimates^51^ of actual species loss. The CFs applied in this study, considering different land-use intensity levels, result in higher losses than in a previous assessment using the countryside SAR approach, and validation of extinctions of endemic mammals, amphibians, and birds demonstrated that the newer CFs perform better.^15^ However, a recent study suggests that the countryside SAR (without land-use intensities) might underestimate losses by 9% at a median level due to overlooked effects of habitat fragmentation.^52^

We proportionally allocate spatially explicit production to domestic consumption and exports according to national data, the standard assumption in current SMRIO studies.^20,53^ Some researchers have attempted nonproportional approaches by incorporating proxy information on the likelihood of export in a region, for example, by assuming a higher likelihood of export where road density is higher than 100 m/km^2^.^17^ Using this as a proxy, we find that 46% of land use within KBAs has a road density higher than this threshold. This means that KBAs are less well connected to the road network than under a proportionality assumption, suggesting that we may slightly overestimate the biodiversity loss from land use within KBAs embodied in trade. However, road densities, as with any other proxy, are yet to be validated at present and there are arguments that it may not be a robust predictor of export proportions (some regions show high export even with a low road density such as areas of the South American Cerrado).^17^ Nevertheless, a nonproportional approach provides another opportunity to reduce uncertainties of SMRIO analysis; thus we need to generate a validated proxy to distinguish domestic consumption from exports in the future.

The methods used here could be combined with other indicators in the future to compare across different approaches. In addition, biodiversity responses are known to be scale-dependent and can be nonlinear (for example, when critical thresholds are reached), making them extremely challenging to incorporate into global models.^54^ Further methodological breakthroughs are needed to represent these dynamics. Biodiversity is itself diverse and multidimensional (involving genetic, species, ecosystem, functional, structural, cultural, and behavioral diversity).^1,38,55,56^ Many species indicators, such as richness, evenness, differentiation, and abundance, have been used to assess biodiversity at multiple scales.^1,38,57,58^ However, indicators going beyond the species level are usually applied in case studies and still need an impact assessment method to be developed for the global scale.^56^ Even though land-use change is the largest single threat to global biodiversity, other threats (e.g., climate change, invasive species, pollution, and overexploitation) can be more important locally and will induce further global biodiversity loss via their interaction.^38,59^ An ongoing challenge is to represent the interaction of these pressures in biodiversity research.^38^ While we only focus on terrestrial species in this study, aquatic species should also be investigated in the future, given their ecological and social importance.

Policymakers have developed many different frameworks for biodiversity protection in multiple different reports.^60−63^ These studies appreciate the impact trade can have on biodiversity loss and have cited recent studies on the topic.^14,18,20,22^ With further efforts to protect biodiversity ongoing, we believe this work presents another useful perspective specifically for key biodiversity areas (KBAs) since KBAs are likely to become the main policy instrument for biodiversity conservation. However, KBAs, despite their importance, are often inadequately protected. Here, we estimated global biodiversity loss driven by human land use within KBAs by combining FABIO and EXIOBASE in an integrated framework with spatial mapping. The integrated framework improves the reliability of studies on environmental impacts related to agriculture and forestry. Our comprehensive assessment can provide guidance for maintaining the integrity of KBAs and global biodiversity.

## Supporting Information Available

Supporting methods with information on product groups in FABIO and EXIOBASE as well as land use datasets; supporting figures about a schematic of the methodology and additional results (PDF)
Data sources and mapping relationships, product sectors and groups in FABIO and EXIOBASE; per-capita and national potential global species loss by countries and products; Spearman’s rank correlation coefficients between species loss and variables determining its magnitude (XLSX)
