# Supplementary material for: Global Human Consumption Threatens Key Biodiversity Areas
Source: Environ Sci Technol. 2022 May 5;56(12):9003–14. doi: 10.1021/acs.est.2c00506 (PMC9228074; doi:10.1021/acs.est.2c00506)
Supplement: Supplementary file 1 — es2c00506_si_001.pdf [file es2c00506_si_001.pdf]

Supporting Information

Global human consumption threatens Key Biodiversity Areas

*Zhongxiao Sun [a, b, *], Paul Behrens [a, c], Arnold Tukker [a, d], Martin Bruckner [e], Laura Scherer [a]*

a. Institute of Environmental Sciences (CML), Leiden University, 2333 CC, Leiden, the Netherlands.

b. College of Land Science and Technology, China Agricultural University, 100193, Beijing, China.

c. Leiden University College The Hague, 2595 DG, The Hague, the Netherlands.

d. The Netherlands Organisation for Applied Scientific Research TNO, 2595 DA, The Hague, the Netherlands.

e. Institute for Ecological Economics, Vienna University of Economics and Business, 1020, Vienna, Austria.

* Zhongxiao Sun. Email: z.sun@cml.leidenuniv.nl

Number of Pages: 17

Number of Supporting Figures: 9

Number of Supporting Tables: 13

Supporting Methods

Product groups

There are 128 agricultural and forestry commodities in FABIO and 172 additional product categories in EXIOBASE. We reported detailed product-based biodiversity loss driven by consumption of FABIO and EXIOBASE in Tables S11 and S12, respectively. For ease of inspection, we classified 200 product categories in EXIOBASE into eight categories (Food, Housing, Transport, Energy, Clothing, Manufacturing, Services, and Other) according to previous work [1]. Food is detailed in FABIO; therefore, we categorized food into ten groups (Grains, Tubers, Vegetables, Fruit, Pulses and nuts, Meat and seafood, Dairy products and eggs, Oils and fats, Sugars, and Stimulants) similar to former studies [2,3]. For the detailed mapping relationship between product categories and reporting groups, see Tables S6 and S7.

Land use datasets

We choose a base year of 2005, which aligns with the characterization factors we employ. To keep the geographic data consistent, we aggregate all land use maps to a common resolution of 5 arcmin.

Cropland: For national cropland, we use the harvested area of 168 types of primary crops from FAOSTAT in 2005 [4] and aggregate them into FABIO's 62 crop sectors. For the spatial maps of cropland, we use 40 categories covering 168 types of primary crops from FAOSTAT at 5 arcmin resolution in 2005, provided by the Spatial Production Allocation Model (SPAM) [5] (see Table S2 for the detailed mapping relationship between FAOSTAT, FABIO, and SPAM crop categories). Specifically, we include the original maps of 42 crop categories , but since "Pearl Millet" and "Small Millet" are not split in FAOSTAT, we aggregate them into millet; similarly, "Arabica Coffee" and "Robusta Coffee" are not split in FAOSTAT, and we aggregate them into coffee. Since FAOSTAT does not report the physical area of crops, we use the ratio of harvested to physical area of crops from SPAM to convert the consumption-based harvested area to the physical area for impact assessment. For national cropland used to produce animal fodder, we use the harvested area derived from FABIO in 2005. However, there is no cropland map of fodder in SPAM. Therefore, we incorporate cropland used to produce animal fodder and calculate it analogously using EarthStat's aggregated fodder maps at 5 arcmin resolution in 2000 [6].

Forest: Previous studies tend to overestimate forest use because they consider all reported forest areas without distinguishing between natural forests and managed or planted forests [7]. Therefore, we link our framework to the latest global forest data at 1 km resolution in 2000 [8]. We assume there are no large changes for the forest map from 2000 to 2005, although this assumption may not hold for some countries [9]. Overall, this may slightly underestimate the effects of forest loss on biodiversity loss. The map downscales forest areas derived from FAO's Forest Resources Assessment into grid cells with two different levels of forest management (Level 1: primary, naturally regrown, and planted forests; Level 2: production, multiple purposes, and other purposes) [8]. First, we use six combinations of forest classes and forest uses as forest use for human production and consumption (Table S4) [8]. After summing the forest area used for production (derived from [8]) in FABIO countries and regions, we allocate the managed and planted forest areas to the sectors "*Wood fuel*", "*Industrial roundwood, coniferous*", and "*Industrial roundwood, non-coniferous*" in FABIO. The allocation uses the share of wood produced by the different sectors in [4]. We then aggregate the forest area map to 5 arcmin, which we use as the uniform spatial resolution in this paper.

Pasture: Pasture was represented by a high-resolution (30 seconds) map from 2005 [10]. We excluded non-productive areas (aboveground NPP below 20 g C m^{-2} yr^{-1}) following a previous study [11,12], and capped the pasture at 100% total land-use coverage in each grid cell.

Infrastructure: We use ESA CCI land cover maps (category Urban Areas at 300 m resolution) in 2005. We assume all infrastructure land is used in final demand (i.e., we assume all infrastructure land only takes part in domestic consumption activities and is not involved in international trade), even though some areas are used for manufacturing sectors. Previous work has outlined the challenges for including infrastructure land more comprehensively [13].

Land-use intensity: For the land-use intensity map, we follow the method provided by [14]. They map the global land system onto five land-use types (we use cropland, pasture, and urban land) with three land-use intensities (minimal, light, intense). A detailed definition of land-use intensity classes is given in Table S3, and detailed conversion rules between Global Land System data and land-use intensity in Table S4. For the definition of forest land-use intensity, see Table S4, which itself is based on [8]. The Global Land System mixes different land-use types within a grid cell. For our purpose, the land-use intensity at a location was judged separately for each land-use type.

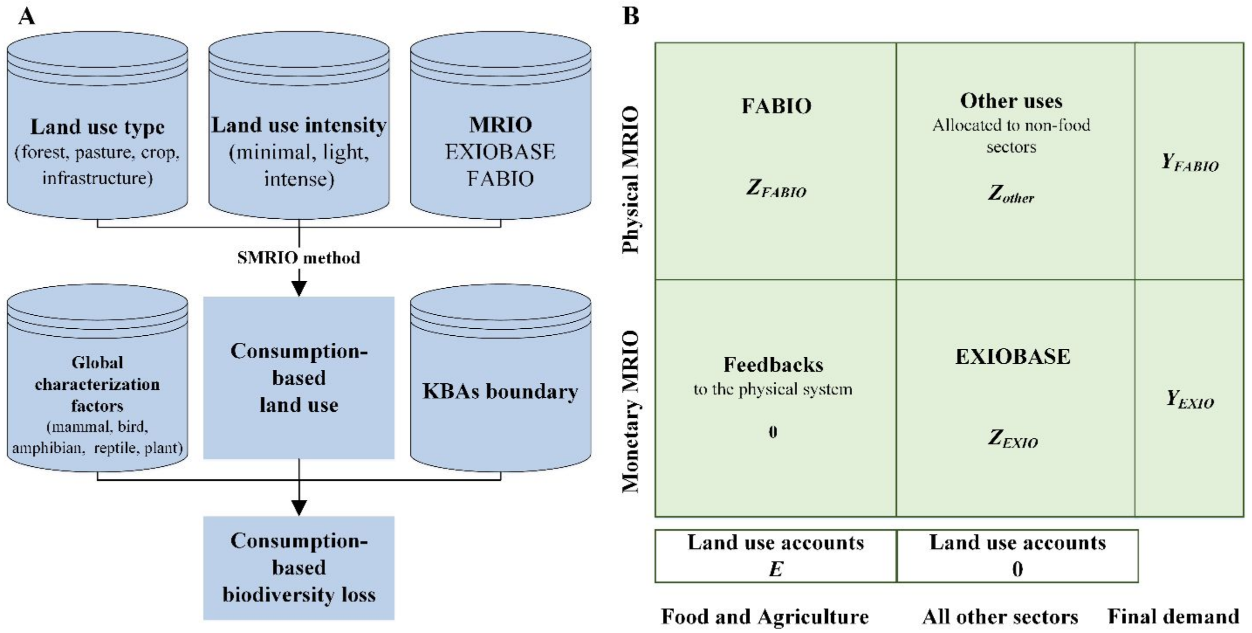

Figure S1. Schematic of the methodology in general (A), and of linking FABIO and EXIOBASE (B).

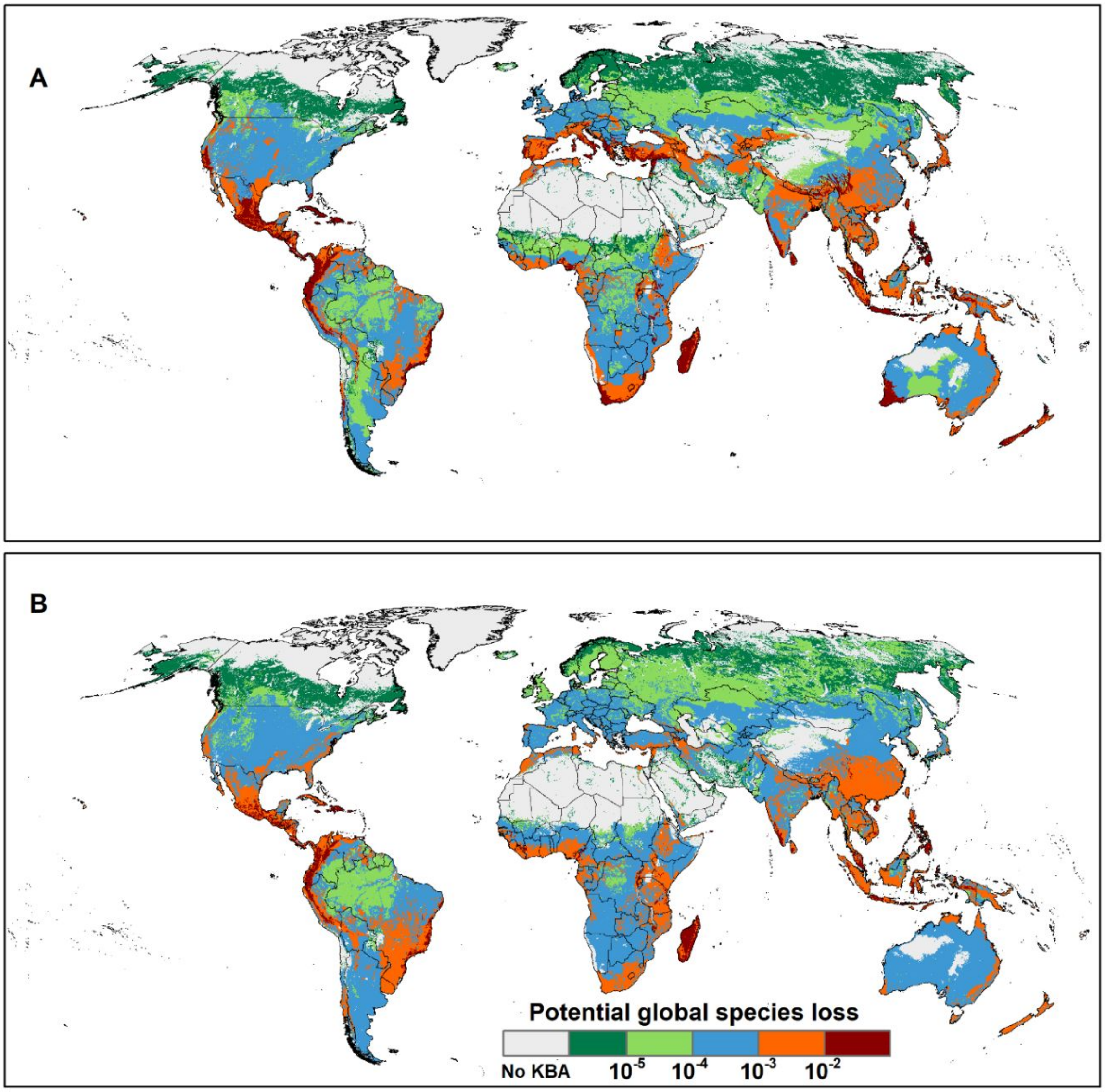

Figure S2. Spatial distribution of potential global species loss driven by land use inside and outside KBAs for A) plants, and B) vertebrates (mammals + birds + amphibians + reptiles). The spatial resolution is 5 arcmin. The results are the sum of potential global species loss across all land-use types and intensities driven by consumption of countries.

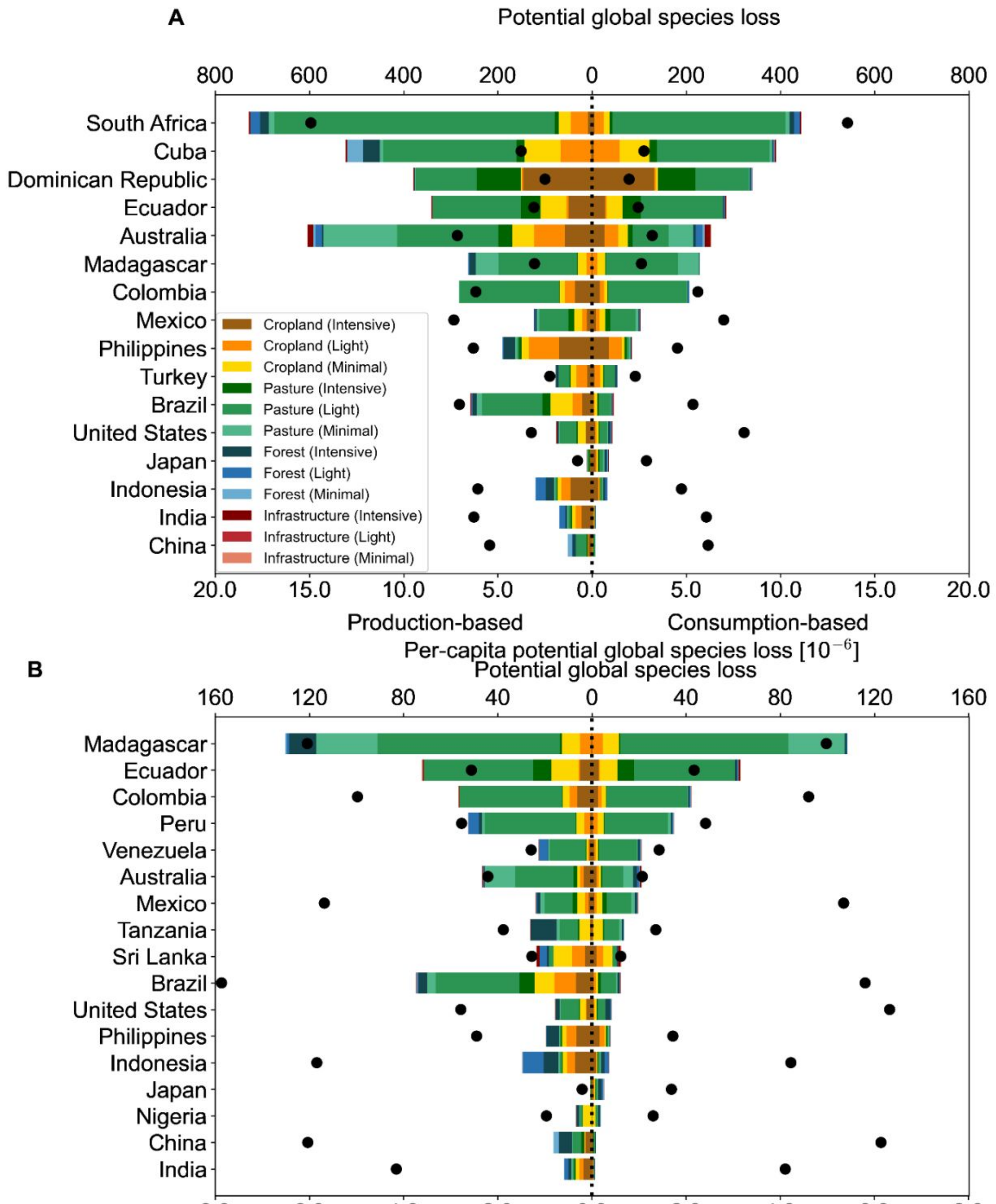

Figure S3. The potential global species loss from land use inside and outside KBAs for plants (A) and vertebrates (B) (mammals, birds, amphibians, and reptiles). On each x-axis (bottom and top of figures), the production-based perspective is shown to the left of zero and the

consumption-based perspective to the right. The y-axis lists the top 15 countries/regions with the largest consumption-based or production-based biodiversity loss from land use within and outside KBAs at the national level. The bar shows the per-capita value of biodiversity loss per land type and land use intensity. The circles show the total national biodiversity loss with a value shown by the upper x-axes on the top of each plot.

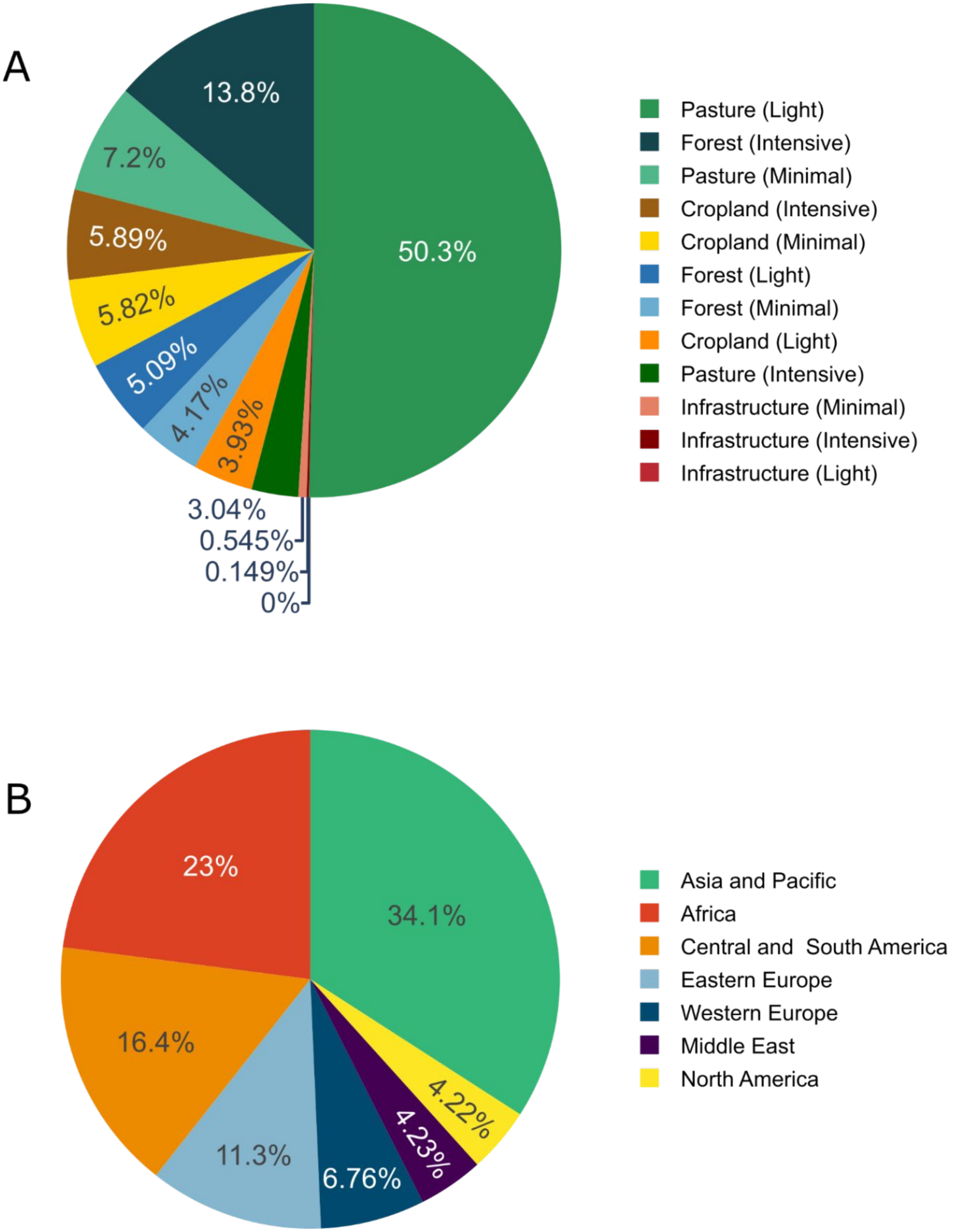

Figure S4. Share of land use within KBAs for different land use types and land use intensities (A) and for different regions (B).

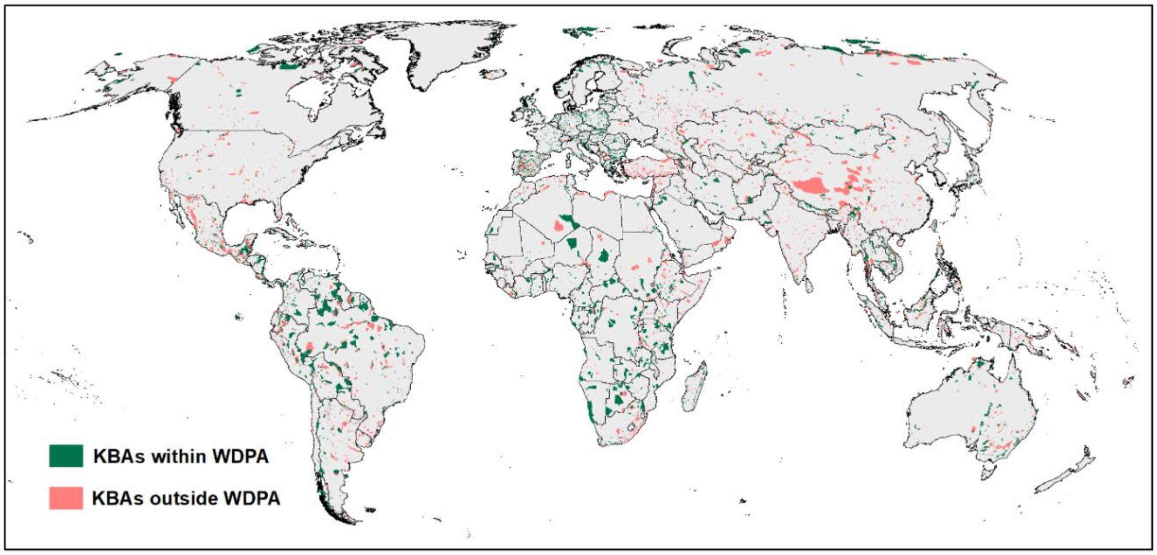

Figure S5. Intersections between KBAs and the World Database on Protected Areas (WDPA).

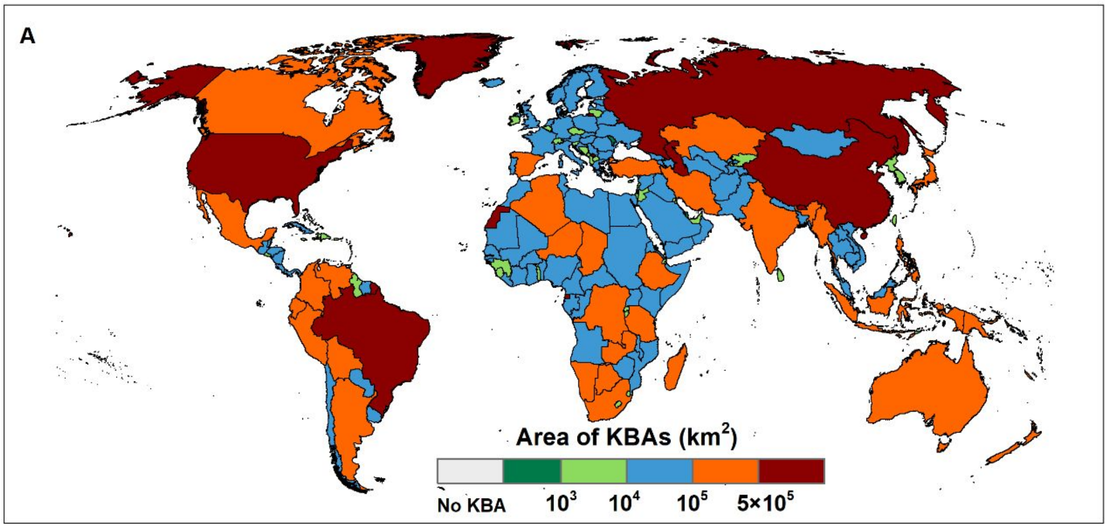

Figure S6. Area of KBAs within countries.

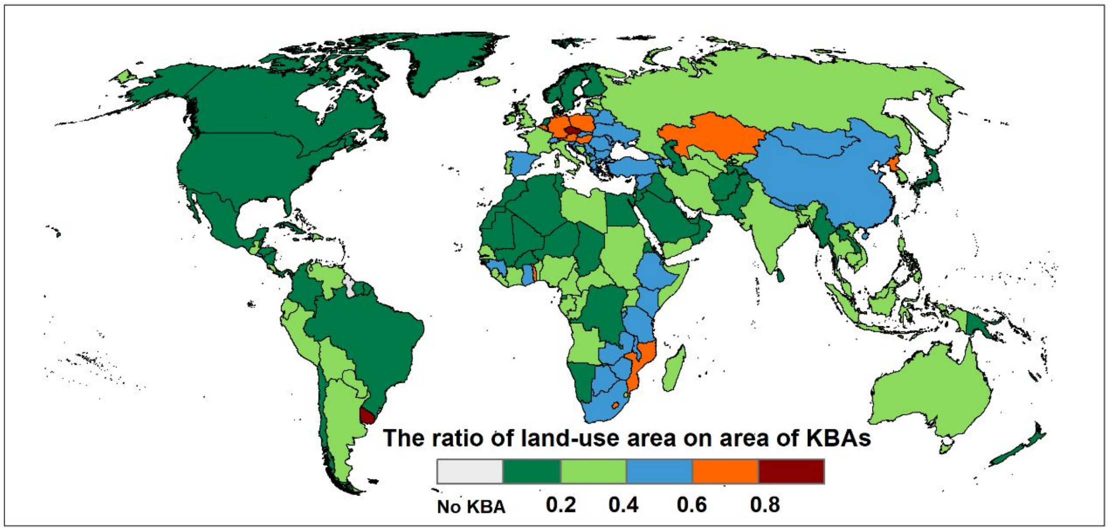

Figure S7. Share of KBAs with anthropogenic land use. The land-use area is all area of human land use within KBAs given a specific country, including all land use types and intensities.

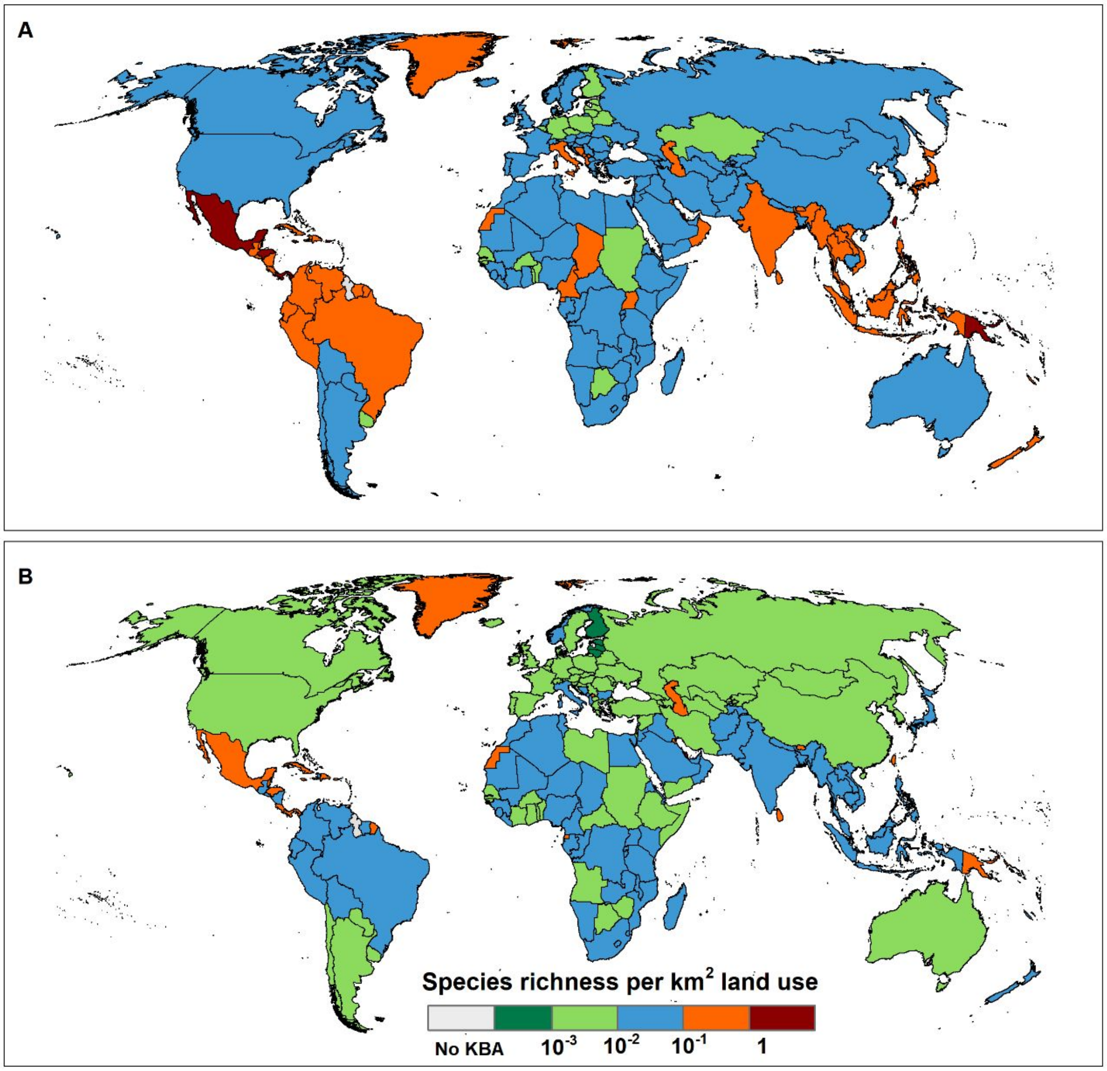

Figure S8. Species richness per area of land use within KBAs for A) plants and B) vertebrates at the country level. The species richness is calculated by multiplying the anthropogenic land use area within KBAs with the species density (i.e. number of species per area). The species density in each pixel is assumed to be the same for all pixels within an ecoregion.

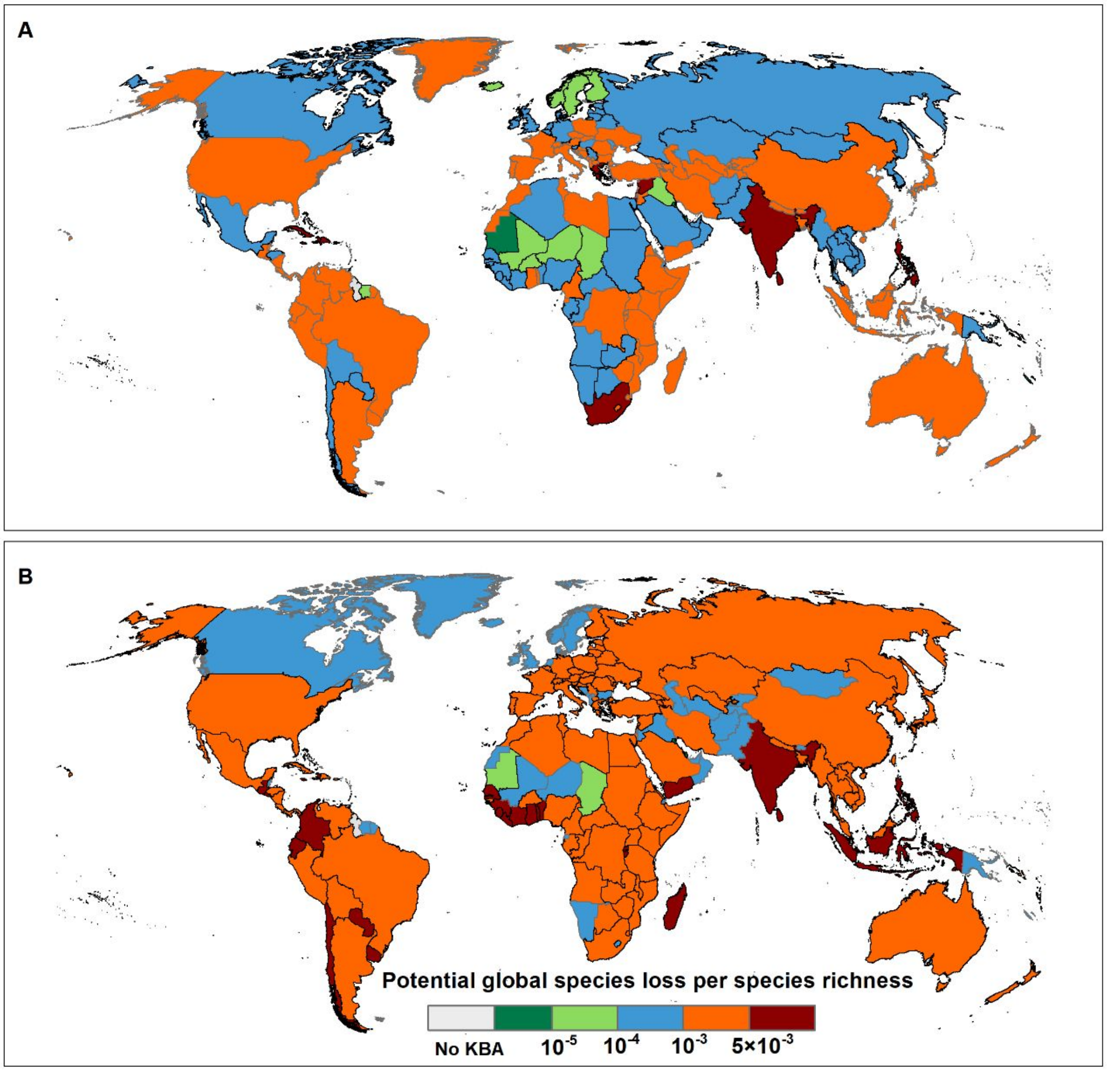

Figure S9. Potential relative global species loss within KBAs due to land use for A) plants and B) vertebrates at the country level.

Dataset S1 (separate file)

Table S1. Data source and spatial resolution of spatial databases used for the paper.

Table S2. The mapping relationship among FAOSTAT sectors, FABIO sectors, and SPAM datasets.

Table S3. Land use classification - description of 16 land use classes used in this study.

Table S4. The mapping relationship between land system classification and land use intensity.

Table S5. Mapping relationship of countries/regions between FABIO, EXIOBASE, and Aggregated regions.

Table S6. The mapping relationship between reporting group and products in FABIO.

Table S7. The mapping relationship between reporting group and products in EXIOBASE.

Table S8. Per-capita production-based and consumption-based potential global species loss from land use within KBAs for all countries (10^{-6} species).

Table S9. National production-based and consumption-based potential species loss from land use within KBAs for all countries (10^{-6} species).

Table S10. National production-based and consumption-based potential global species loss from land use inside and outside KBAs for all countries (10^{-6} species).

Table S11. Consumption-based potential global species loss from land use within KBAs due to final consumption in FABIO (10^{-6} species).

Table S12. Consumption-based potential global species loss from land use within KBAs due to final consumption in EXIOBASE (10^{-6} species).

Table S13. Spearman's rank correlation coefficient between species loss and its drivers.

References

(1) Scherer, L.; Behrens, P.; de Koning, A.; Heijungs, R.; Sprecher, B.; Tukker, A. Trade-Offs between Social and Environmental Sustainable Development Goals. *Environ. Sci. Policy* **2018**, *90*, 65–72. https://doi.org/10.1016/j.envsci.2018.10.002.

(2) Semba, R. D.; de Pee, S.; Kim, B.; McKenzie, S.; Nachman, K.; Bloem, M. W. Adoption of the 'Planetary Health Diet' Has Different Impacts on Countries' Greenhouse Gas Emissions. *Nat. Food* **2020**, *1* (8), 481–484. https://doi.org/10.1038/s43016-020-0128-4.

(3) Behrens, P.; Kiefte-De Jong, J. C.; Bosker, T.; Rodrigues, J. F. D.; De Koning, A.; Tukker, A. Evaluating the Environmental Impacts of Dietary Recommendations. *Proc. Natl. Acad. Sci. U. S. A.* **2017**, *114* (51), 13412–13417. https://doi.org/10.1073/pnas.1711889114.

(4) FAOSTAT. FAOSTAT.

(5) You, L.; Wood-Sichra, U.; Bacou, M.; Koo, J. *Spatial Production Allocation Model (SPAM) 2005 v3.2*; 2017.

(6) Monfreda, C.; Ramankutty, N.; Foley, J. A. Farming the Planet: 2. Geographic Distribution of Crop Areas, Yields, Physiological Types, and Net Primary Production in the Year 2000. *Global Biogeochem. Cycles* **2008**, *22* (1), n/a-n/a. https://doi.org/10.1029/2007GB002947.

(7) Bruckner, M.; Fischer, G.; Tramberend, S.; Giljum, S. Measuring Telecouplings in the Global Land System: A Review and Comparative Evaluation of Land Footprint Accounting Methods. *Ecol. Econ.* **2015**, *114*, 11–21. https://doi.org/10.1016/J.ECOLECON.2015.03.008.

(8) Schulze, K.; Malek, Ž.; Verburg, P. H. Towards Better Mapping of Forest Management Patterns: A Global Allocation Approach. *For. Ecol. Manage.* **2019**, *432*, 776–785. https://doi.org/10.1016/J.FORECO.2018.10.001.

(9) Hansen, M. C.; Potapov, P. V.; Moore, R.; Hancher, M.; Turubanova, S. A.; Tyukavina, A.; Thau, D.; Stehman, S. V.; Goetz, S. J.; Loveland, T. R.; et al. High-Resolution Global Maps of 21st-Century Forest Cover Change. *Science (80-.).* **2013**, *342* (6160), 850–853. https://doi.org/10.1126/science.1244693.

(10) Hoskins, A. J.; Bush, A.; Gilmore, J.; Harwood, T.; Hudson, L. N.; Ware, C.; Williams, K. J.; Ferrier, S. Downscaling Land-Use Data to Provide Global 30″ Estimates of Five Land-Use Classes. *Ecol. Evol.* **2016**, *6* (9), 3040–3055. https://doi.org/10.1002/ece3.2104.

(11) Erb, K.-H.; Gaube, V.; Krausmann, F.; Plutzar, C.; Bondeau, A.; Haberl, H. A Comprehensive Global 5 Min Resolution Land-Use Data Set for the Year 2000 Consistent with National Census Data. *J. Land Use Sci.* **2007**, *2* (3), 191–224. https://doi.org/10.1080/17474230701622981.

(12) Marques, A.; Martins, I. S.; Kastner, T.; Plutzar, C.; Theurl, M. C.; Eisenmenger, N.; Huijbregts, M. A. J.; Wood, R.; Stadler, K.; Bruckner, M.; et al. Increasing Impacts of Land Use on Biodiversity and Carbon Sequestration Driven by Population and Economic Growth. *Nat. Ecol. Evol.* **2019**, *3* (4), 628–637. https://doi.org/10.1038/s41559-019-0824-3.

(13) Sun, Z.; Tukker, A.; Behrens, P. Going Global to Local: Connecting Top-Down Accounting and Local Impacts, A Methodological Review of Spatially Explicit Input–Output Approaches. *Environ. Sci. Technol.* **2019**, *53* (3), 1048–1062. https://doi.org/10.1021/acs.est.8b03148.

(14) Newbold, T.; Hudson, L. N.; Hill, S. L. L.; Contu, S.; Lysenko, I.; Senior, R. A.; Börger, L.; Bennett, D. J.; Choimes, A.; Collen, B.; et al. Global Effects of Land Use on Local Terrestrial Biodiversity. *Nature* **2015**, *520* (7545), 45–50. https://doi.org/10.1038/nature14324.
